# How to make predictions about future infectious disease risks

**DOI:** 10.1098/rstb.2010.0387

**Published:** 2011-07-12

**Authors:** Mark Woolhouse

**Affiliations:** Centre for Infectious Diseases, University of Edinburgh, Ashworth Laboratories, Kings Buildings, West Mains Road, Edinburgh EH9 3JT, UK

**Keywords:** animal health, emerging diseases, good practice, modelling, public health, risk factors

## Abstract

Formal, quantitative approaches are now widely used to make predictions about the likelihood of an infectious disease outbreak, how the disease will spread, and how to control it. Several well-established methodologies are available, including risk factor analysis, risk modelling and dynamic modelling. Even so, predictive modelling is very much the ‘art of the possible’, which tends to drive research effort towards some areas and away from others which may be at least as important. Building on the undoubted success of quantitative modelling of the epidemiology and control of human and animal diseases such as AIDS, influenza, foot-and-mouth disease and BSE, attention needs to be paid to developing a more holistic framework that captures the role of the underlying drivers of disease risks, from demography and behaviour to land use and climate change. At the same time, there is still considerable room for improvement in how quantitative analyses and their outputs are communicated to policy makers and other stakeholders. A starting point would be generally accepted guidelines for ‘good practice’ for the development and the use of predictive models.

## 1. Introduction

This review is concerned with three kinds of predictions. The first kind is to do with the risk that an exotic or novel infection will appear in a given host population. This risk has been formally estimated for, for example, rabies or foot-and-mouth disease (FMD) being introduced into the UK [1,2]. The second kind is that, given an infectious disease is present, how fast will it spread, how many people or animals will be affected and how long will it persist for? This has been attempted for a wide variety of infectious diseases including Aquired Immune Deficiency Syndrome (AIDS) [3], bovine spongiform encephalopathy (BSE) [4] and FMD [5]. The third kind of prediction is to do with what might happen if an intervention is attempted: if people or animals are to be treated, vaccinated or quarantined in an attempt to contain an epidemic then how many, how selected and how quickly? There is a substantial literature addressing this type of question, e.g. FMD [6] or human influenza [7].

The review will consider the need for quantitative models (§2), the kinds of approaches available (§3), data requirements (§4) and applications to the emergence of novel infectious diseases (§5). Recurring themes are the need for quantitative analyses that account for the nonlinear dynamics of infectious diseases, the links between models and data, the importance of communication with end users (especially policy makers) and the inter-disciplinary nature of the research agenda needed to improve predictive capacity in the future. The focus here is mainly on infectious diseases of humans and animals, although many of the issues are equally relevant to plant diseases, as has been discussed elsewhere [8,9].

## 2. Role of quantitative models

**Figure 1.**
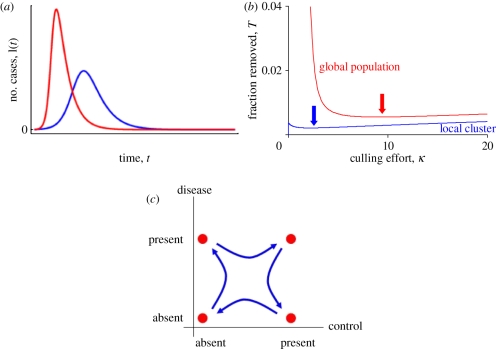
Non-linearities in infection dynamics. (*a*) Force-of-infection and duration of an outbreak. Reduced force-of-infection can *increase* the expected duration of an outbreak, as illustrated by two numerical realizations of the standard susceptible–latent–infectious–recovered (SLIR) model [3]. Both have mean latent period = 1 time unit and mean recovery period = 1 time unit but the *per capita* transmission rate is halved from high (red) to low (blue), resulting in a smaller but longer lasting outbreak. (*b*) Impact of pre-emptive culling. Analysis of the impact of increased pre-emptive culling effort on the total loss of livestock farms during a FMD epidemic. Fraction of the global population removed (red line) and fraction of the global population removed within a single local cluster of 50 farms (blue line) are shown as functions of the number of pre-emptive culls per case. Parameter values used approximate those for the 2001 UK FMD epidemic. The culling effort minimizing global losses (red arrow) is almost 4× higher than that minimizing local losses (blue arrow). Figure re-drawn from [10]. (*c*) Relationship between the presence of disease and the implementation of control. A local host population is shown moving (blue arrows) in sequence between four states (red dots): first, disease is introduced; then control is implemented; then disease is eliminated; then control ceases. This describes the expected sequence of events when control is implemented reactively and locally. Depending on how many local populations are in each of the four states at a given time point, a cross-sectional study could generate a positive or zero correlation between levels of disease and control effort as easily as a negative one (the naive expectation), even if control is fully effective.

Making the kinds of predictions listed in §1 requires some kind of ‘model’, even if this is only a mental model based on previous experience, expert opinion or a back-of-the-envelope calculation. For some questions, this may be adequate; an example is given below (§3*a*). For other questions, these informal approaches can be highly unreliable, the reason being that infectious diseases have nonlinear dynamics. One way to express this is that the biggest risk factor for acquiring an infection is the presence of infectious individuals, which introduces positive feedback into epidemic processes, in turn making the expected trajectory of an epidemic or the likely impact of control measures considerably more difficult to predict than is the case for non-communicable diseases such as stroke, cancer or obesity. On occasion, these nonlinearities can make infectious disease dynamics counterintuitive: some examples are shown in figure 1.

The first example (figure 1*a*) illustrates the simple but important observation that decreasing the transmission rate (achieved, for example, by more rapid quarantining of cases) does, as might be expected, reduce the size of an outbreak but may increase rather than decrease the duration of the outbreak. When some of the major consequences of an epidemic are indirect—such as closure of schools, restrictions on travel or trade or loss of tourism revenue—and reflect only the presence of disease rather than the absolute numbers of cases, then the increased duration represents a serious problem.

The second example (figure 1*b*) is rather more complicated and has been the subject of detailed analysis [10]. This concerned optimal levels of pre-emptive culling of livestock on farms in the neighbourhood of a FMD case, where ‘optimal’ implies minimizing the total number of farms lost over the course of an epidemic. The analysis identified a conflict between local and global optima. Using parameter values consistent with the UK 2001 FMD epidemic, the globally optimal culling rate is almost four times as high as the local optimum; this reflects the need to reduce the likelihood of disease spreading from one local cluster to others. From a local perspective, this level of culling might well be regarded as ‘overkill’, ‘draconian’ or ‘disproportionate’ (all terms that were used during 2001), but the local perspective is not the most relevant one for decision makers. Another result was to illustrate that under-control is more dangerous than over-control, ultimately leading to more farms lost (the gradient of the curves are much steeper to the left of the optima than to the right). This result is quite general: if control measures are inadequate then an epidemic will not be brought under control and more of the population will be affected in the long run. If, as will often be the case in practice, there is uncertainty about the optimal level of control effort then it will often be preferable to err towards too much rather than too little.

The third example (figure 1*c*) concerns the relationship between causes and effects. Intuitively, it might be expected that statistical analysis would indicate a negative relationship between disease incidence and control effort (at least, if control was effective). Although this would normally be the case when control is implemented proactively and uniformly, it is not necessarily the case when control is implemented reactively and locally. In those circumstances disease must be present before a control effort is initiated, disease and control will then co-occur until disease has been locally eliminated, at which point control will cease. This sequence of events is depicted in figure 1*c* and is as likely to produce a positive correlation or no correlation as a negative one. This is a well-known paradox that applies in other contexts too [11], but misinterpretations persist, e.g. of the effectiveness of control by pre-emptive culling during the UK FMD 2001 epidemic [12,13]. This issue is a good illustration of the dangers of static analyses of dynamic systems, and is a strong argument for the use of dynamic models in infectious disease epidemiology.

## 3. Approaches to prediction

Whatever methodology is used, a key challenge in making any kind of prediction is to establish the extent to which the past is likely to be an accurate guide to the future. There are different levels of predictability that may be taken to apply, expressed here in terms of the structure and parameter estimates of a mathematical model fitted to a previous FMD epidemic but now having to be adapted to make predictions about a new epidemic.

- No change. The input data, parameter values and the model itself are all judged to be applicable to the new epidemic.
- The input data change. As a simple example, there may be changes to the location, size and species composition of livestock farms. Such changes, if known, would be readily incorporated into a new model.
- The parameter values change. This would be the case if, for example, a different strain of FMD virus was introduced, perhaps with different transmission characteristics. Or if farming practices had altered in ways, such as improvements in biosecurity, which changed FMD transmission rates. These kinds of changes would be difficult to quantify *a priori*, and new parameter values may need to be estimated from early epidemic data.
- The model changes (i.e. the original model structure/assumptions are incorrect for the new epidemic). This could be the case if, for example, the strain of FMD virus introduced was much more liable to airborne transmission, requiring that this feature be built into the models.

Addressing (ii) is straightforward, (iii) is reliant on methods for rapid estimation and re-estimation of parameter values as data accumulate (see, for example, [14] for a state-of-the-art application), but (iv) will most probably depend on timely input from disease experts.

In practice, various approaches have been used for making predictions about future disease risks. These include: expert opinion, statistical methods, simulation modelling, and risk modelling.

### (a) Expert opinion

There is a now a substantial literature on methodologies for systematically surveying expert opinion (e.g. [15]). One example of their application to future infectious disease risks derives from a 2006 UK government Foresight project (see [16] for details). There were two components to this study: the identification and ranking of future disease risks (or, more correctly, ‘hazards’) and the identification of factors involved in changes to these risks in the future (so-called ‘drivers’ of risk).

As with all studies of this nature, the results necessarily reflect the expertise, interests and geographical locations of the participants as well as, crucially, the precise questions they were asked. The main hazards identified are listed in table 1. The two most consistent concerns of the experts involved were the emergence of novel pathogens and of drug-resistant variants of existing pathogens. There was much less agreement on the importance of different drivers of changes in infectious disease risks, presumably in part reflecting much more variability in these across different disease systems but also reflecting genuine uncertainty as to what the main drivers will be. Climate change, for example, was much more of a concern for infectious diseases in Africa than in the UK, and for 2030 rather than 2015. Similarly, economic and social factors were seen as much more important drivers for human infectious disease risk in Africa than in the UK. The study had many limitations [16], not least the simplistic assumption that drivers will act independently on disease risks, but nonetheless it provides a useful starting point for more evidence-based approaches (see §5).

**Table 1.** Main infectious disease hazards to humans, animals and plants globally as identified by a 2006 UK Foresight study [16].

(1)	new pathogen species and novel variants
(2)	pathogens acquiring resistance
(3)	the ‘Big Three’: HIV/AIDS, TB, malaria
(4)	acute respiratory infections
(5)	sexually transmitted infections
(6)	zoonoses
(7)	transboundary animal diseases
(8)	epidemic plant diseases

### (b) Statistical methods

The statistical workhorse of risk factor analysis is the generalized linear model (GLM) [17,18]. The methodology is well established and its application routine, but it has its limitations for the analysis and prediction of infectious disease data. As discussed in §2, the risks of individuals becoming infected are dynamic and not independent of one another; this introduces spatial and temporal autocorrelations that may be difficult to account for [19]. This problem is exacerbated when the intention is to predict future risks, perhaps under different scenarios such as a range of possible intervention strategies. Extrapolations of statistical models have limited value in this context. Hence, as discussed below, dynamic models have often been preferred.

Making epidemiological predictions requires knowledge of both the risk of infection but also, crucially, the risk of transmitting infection if infected. The scientific literature contains many thousands of studies of the former but far less attention has been paid to the latter (see [20] for an example). Sometimes susceptibility and transmission may be closely related, e.g. for vector-borne diseases, but for others they may be quite distinct, e.g. for faecal–oral transmitted infections. There are some obvious reasons for this lack of attention. First, it is often much harder to establish that an individual has transmitted infection than that it has acquired infection, whether this information is to be inferred from contact tracing studies (as carried out during the 2001 UK FMD epidemic [21]) or from the analysis of pathogen typing or sequence data (as carried out during the 2007 UK FMD outbreak [22]). Second, the sample size available is not the total population but the infected population (each of whom may or may not have transmitted infection onwards), typically a much smaller number with a corresponding loss of statistical power to identify risk factors.

**Figure 2.**
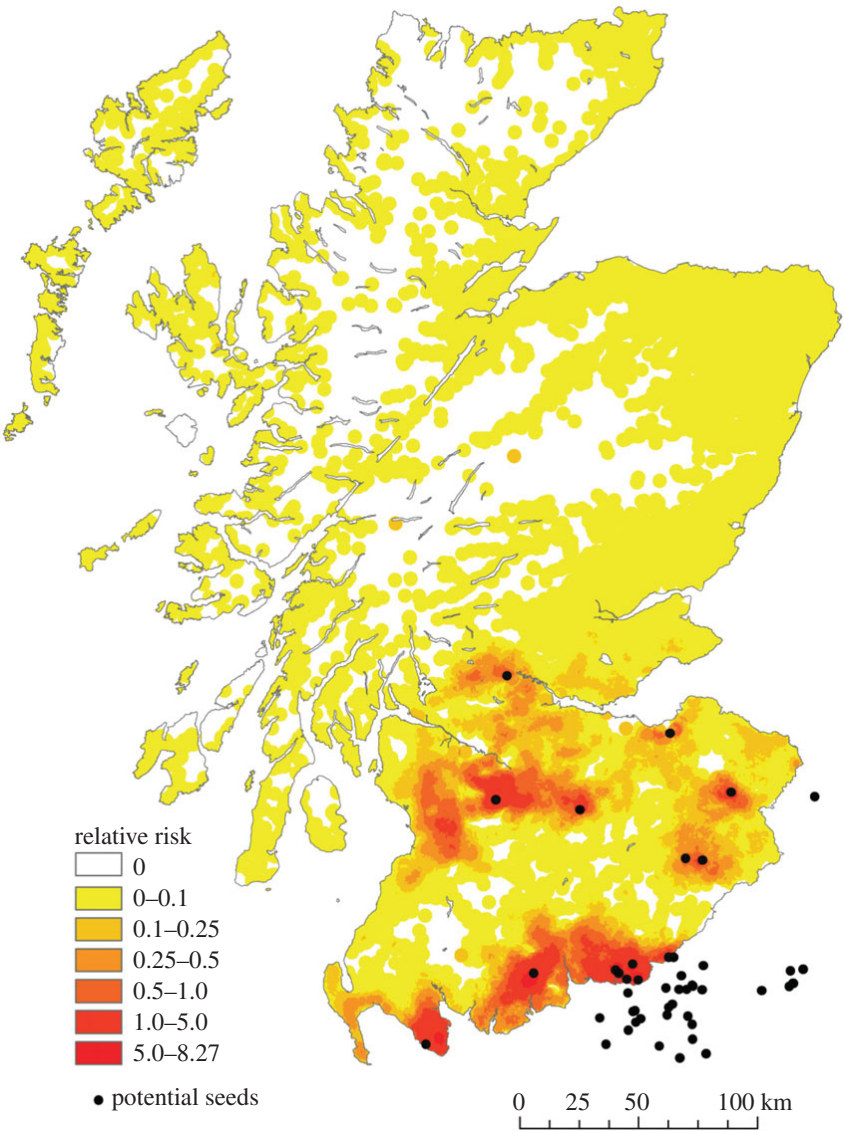
Estimated risk of FMD in Scotland in September 2007. Black dots represent ‘at risk’ farms linked (directly or indirectly) by livestock movements to the FMD-affected region in Surrey. Colour scale shows the relative risk of secondary cases if FMD were present in the ‘at risk’ farms, as derived from an analysis of risk factors using data from the UK 2001 FMD epidemic [23].

The outputs of a risk factor analysis are routinely expressed in terms of odds ratios associated with each of the risk factors in the model, identifying the main drivers of risk in the study population. Often, however, it is also useful to calculate the risk (e.g. the probability of being infected) for each individual in the population, based on their individual risk factors (e.g. [19]). A risk profile or risk map is thus generated which can be used for direct surveillance, prevention or control efforts. An example of this occurred in Scotland in September 2007, when there was a FMD outbreak in Surrey in England [23]. A risk map was generated based on livestock movement records and local risk factors, and this was used to direct surveillance efforts, allowing Scotland to provide evidence of freedom from disease much more quickly than would otherwise have been possible, and so accelerating the lifting of movement restrictions imposed in response to the risk of FMD (figure 2).

### (c) Risk modelling

Here, risk modelling is defined as the formal, quantitative estimation of the probability of specified adverse effects from defined hazards [15]. A variety of approaches can be used in risk modelling, sometimes in combination for complex problems.

Indeed, in practice, applications to specific problems often require a bespoke quantitative analysis. For example, estimation of the risk of rabies entering the UK through imported pets (expressed as the mode and distribution of the number of years between rabies entries) [1] or the risk of FMD entering the UK through illegally imported meat products (partitioned by geographical origin of the imports) [2]. In principle, similar kinds of approaches could be applied to other kinds of question too; for example, estimating the probability of the emergence of a novel pathogen (see §5), or the probability that introduction of a pathogen will lead to an epidemic.

A significant challenge to risk modellers (whatever methodology is used) is accurate communication of the results, particularly to decision makers who may be unfamiliar with the technical details of the analyses [24,25]. It is now widely accepted that formal measures of uncertainty (typically 95% confidence/credible intervals or a full depiction of the range of outputs obtained) must be linked to the ‘headline’ result. Issues of verification and validation (discussed in more detail below) also need to be addressed.

### (d) Dynamic or process modelling

In the past decade, there has been a shift away from the deterministic differential equation models—sometimes referred to as mean field models—that were the foundation of epidemiological modelling for almost a century [3]. Much recent modelling work on both human and animal diseases has used stochastic, individual-based models (IBMs; see [26] for an overview). This approach has a number of advantages:

— individuals are represented explicitly and their infection status or any other attribute can be tracked;
— heterogeneities between individuals (attributes such as age or spatial location, or risk factors for infection) can be represented directly and non-parametrically (i.e. no assumptions need to be made about statistical patterns of variation across the population); and
— complex individual histories, e.g. with respect to interventions, can be incorporated in a fully realistic manner.

There are also well-recognized disadvantages:

— when tailored to specific events, the models may lack generality;
— IBMs are often very complex and cannot be studied analytically, making it hard to understand the relationship between inputs and outputs;
— the models can be hard to parametrize and efforts to formally fit IBMs to complex epidemic data, e.g. for FMD in the UK in 2001, have met with only qualified success [27,28]; and
— the models may require a large amount of very detailed input data, e.g. individuals' travel patterns, which may be difficult to acquire [7].

The above issues underline the importance of model verification and model validation. Verification is demonstration that the model accurately represents the developer's conceptual description and specifications, i.e. that it does what it is supposed to do. Validation means simply that the model achieves pre-set performance requirements, i.e. that it is fit for purpose. Verification and validation are essential features of good practice in the development and the use of mathematical models [29,30] especially, of course, when the models are intended to be policy-relevant.

The scientific literature tends to treat dynamic modelling and statistical risk analysis as distinct activities, but recently there have been attempts to relate the two approaches to one another. One study [19] compared the ‘accurary’ (a measure of the correspondence between estimated risk and actual outcome) of a logistic regression model and a previously published IBM-based analysis [31], in the context of predicting which farms were at risk of being infected with FMD during the UK 2001 epidemic. The statistical approach performed slightly better using this measure, suggesting possible refinements to the simulation model. Another study [32] compared statistical risk factor analysis and stochastic simulation models fitted using maximum-likelihood methods, as applied to *Escherichia coli* O157 on cattle farms. Reassuringly, the two approaches agreed well in terms of identifying which risk factors were important and which farms were at most risk of infection.

## 4. Data inputs

The kinds of data inputs required to make predictions about future disease risks can usefully be divided into those concerning the disease, the host or the environment.

### (a) Disease

Disease data fall into two categories. First, there are data concerning the ‘natural history’ of infection, including the latent, incubation and infectious periods [33]. Again there is an asymmetry in research effort, here between studies addressing pathogenesis (how the pathogen affects the host and vice versa) and studies addressing transmission of the pathogen from one host to another. For example, it is often unclear whether the latent period (time from exposure to becoming infectious) is longer or shorter than the incubation period (time to showing clinical symptoms or signs), yet this basic information is crucial when control efforts depend on detecting infection on the basis of clinical observation, as is the case for many epidemic diseases including influenza, severe acute respiratory syndrome (SARS) and FMD.

Second, there are surveillance data describing the distribution and spread of infection through space and time. Correct interpretation of these data requires knowledge of the sensitivity and specificity of the diagnostic test used, and of reporting patterns (especially under-reporting and reporting delays). Under-reporting is a major problem for diseases such as human influenza or sheep scrapie, where the great majority of cases may be unrecognized and unreported. This was also the case during the early stages of the BSE epidemic [4]. This may mean that actual infection rates are orders of magnitude higher than indicated by reported case numbers (e.g. pandemic H1N1 in England in 2009 [34]). Reporting delays can also obscure epidemic dynamics in real time. Without a detailed understanding of reporting patterns, case data (and, no less, indirect measures such as Internet search behaviour [35]) become unreliable inputs into quantitative analysis and need to be supplemented or replaced, e.g. by active case finding or serosurveillance (looking for evidence of exposure to infection based on detection of specific antibody responses).

### (b) Host

Knowledge of host demography is an essential component of any epidemiological analysis. Key demographic variables typically include the age and sex structure of the population and its density and spatial distribution. In many instances these may not be known precisely, raising the question of how good is good enough? This issue has been addressed in detail for the specific issue of the potential spread of FMD between cattle farms in the USA where the behaviour of simulated epidemics was compared given different levels of precision regarding farm location, allowing specification of the level of spatial resolution required to make robust recommendations regarding control strategies [36].

Another key issue for disease spread is patterns of host movement. These have been extensively studied for livestock for which, in the UK and elsewhere, comprehensive records of movements of individual or batches of animals between agricultural holdings have been available for more than a decade [37,38]. The availability of high-quality movement data makes it much easier to identify movement as a risk factor, but for some infections, such as *E. coli* O157, other, harder-to-quantify factors (relating to wider environmental contamination) may be of much greater epidemiological significance [39,40]. For humans, although information on specific kinds of movements such as commuting distances or international air travel is often available, these sources provide at best an incomplete and potentially a biased picture of relevant human movement patterns. In the case of wildlife, data on migratory patterns have been used as inputs into risk analysis, for example, for the spread of avian influenza internationally by wildfowl [41].

These aspects of host demography and movements, together with other aspects of host phenotype (e.g. behaviour, infection history and immune status) and sometimes genotype, make the host population heterogeneous with respect to the potential for the spread of infectious diseases. These heterogeneities can have a major impact on disease dynamics, and need to be recognized and quantified to assess properly the risk of spread of infection and the potential impact of interventions [42]. One example is the influence of spatial heterogeneity in livestock densities in the UK on the potential spread of FMD [43]: in 2001, the disease reached areas of high livestock densities, resulting in a major epidemic; in 2007, it remained confined to low-density areas and spread was, as predicted at the time, limited.

A common confounding issue is the involvement of additional host populations, be they reservoirs of infection (such as badgers for bovine tuberculosis or wildfowl for H5N1 influenza), or vectors (such as culicoides midges for bluetongue virus). Typically, much less demographic information is available for reservoirs (especially wildlife reservoirs) and vector populations, which may constrain predictive analysis.

### (c) Environment

Here, ‘environment’ is taken to refer to any factor that is not an attribute of the pathogen or the host(s). This encompasses a vast range of possible influences on disease dynamics, ranging from levels of hygiene in hospitals to land use and climate, including factors that influence disease vectors or intermediate hosts, or those that influence reservoir host populations.

Climate has commonly been investigated as a possible environmental driver of disease risk. Examples include associations of climate-related factors with outbreaks of African horse sickness [44], cholera [45] and Rift Valley fever [46] or shifts in endemic levels of malaria infection [47]. Climate change is an important and topical subject, but that is not its only attraction for studies to predict changes in disease risk. Climate data are quantitative, measured at fine spatial scales and, crucially, sophisticated projections are available describing how climate variables are expected to change over coming decades. A few other drivers, such as human demography (population density, age structure, urbanization, etc.) have similar attributes. Many others (such as investment in public health, population displacement, natural disasters, war, etc.) may well be just as important but their role is hard to quantify and their future behaviour even harder to predict [16]. In short, identifying drivers of disease risk and predicting their future impact are very much the art of the possible. As an example, two events that could have major impacts on the future of livestock disease in European countries are reform of the Common Agricultural Policy (CAP)—which would greatly influence farming practices—and the accession of countries such as Turkey that regularly experience exotic infections such as FMD. These events are extremely hard to ‘predict’, in contrast to the way that climate change is predicted, but may well turn out to have considerably greater epidemiological consequences over the next few decades.

Disease prevention programmes and/or reactive control efforts are, of course, key ‘environmental’ drivers of the dynamics of many infections at a variety of spatial and temporal scales, and may vary for many reasons, including shifts in policy objectives [48]. Some kinds of intervention are relatively easy to quantify and evaluate: examples include the impact of vaccination programmes and the role of herd immunity [49], or the impact of culling for the control of animal diseases [50]. For other interventions—such as biosecurity measures against FMD, or the use of face masks against influenza—their use is not reliably reported nor their efficacy known. Another difficulty is knowing the way in which control measures are applied in practice. For example, the pre-emptive culling during the UK 2001 FMD epidemic was found retrospectively to have been significantly biased towards lower risk farms (mostly smaller sheep farms) rather than higher risk farms (mostly larger farms with cattle). The actual impact of the culling programme is, therefore, likely to have been less than anticipated when the policy was first proposed [51].

## 5. Emerging infectious diseases

An important, topical and challenging application of methods to predict disease risk concerns the emergence of novel pathogens. Pathogens are emerging and re-emerging at alarming rates [52]. Numerous studies of specific pathogens have linked their emergence/re-emergence to particular drivers (table 2), although most such studies are purely descriptive and the association with drivers is based on no more than a subjective interpretation of events. Better evidence is needed to make strong assertions about cause and effect:

— individual drivers need to be identified more precisely than high-level descriptors such as ‘climate change’;
— drivers need to be measured and mapped, and changes in drivers need to be monitored;
— interactions between multiple drivers need to be identified;
— statistical associations between emergence events and drivers or combinations of drivers need to be tested; and
— ideally, functional relationships between emergence events and drivers need to be demonstrated.

**Table 2.** The 10 most frequently cited drivers of the emergence and re-emergence of infectious diseases [53].

(1)	changes in land use or agricultural practices
(2)	changes in human demographics and society
(3)	poor population health
(4)	hospitals and medical procedures
(5)	pathogen evolution
(6)	contamination of food sources or water supplies
(7)	international travel
(8)	failure of public health programmes
(9)	international trade
(10)	climate change

At present, however, there are very few examples of systematic studies of the relationship between emergence and drivers of emergence. Jones *et al.* [54] carried out a literature survey of emerging disease events (defined as the first reported appearance of a previously unknown pathogen species or strain, including drug-resistant strains) since the 1940s and attempted to relate these to a set of possible drivers using multi-variate logistic regression. They found associations between emergence events and drivers such as human population density and growth rate, latitude, rainfall and biodiversity. The main challenge was fully accounting for highly suspect reporting patterns, with fully one-third of all reported events occurring in the USA and very few in countries such as China, India, the Philippines or Mexico [55]. An interesting conclusion from this study was to suggest the existence of emerging disease ‘hotspots’, an important concept that will no doubt be refined by further analysis in the future.

The same study [54] also suggested that emergence events have increased in frequency through time. Again, this result is sensitive to the robustness of their reporting bias correction, but it echoes the more descriptive notion of a ‘perfect storm’ for emerging diseases in the early twenty-first century (L. King 2005, personal communication). This idea reflects the view that many drivers of emergence (table 2) are changing in ways that, at least intuitively, should promote the emergence of novel pathogens. Even so, there are no hard data to compare on trends in emergence rates prior to the past few decades. Nonetheless, it is reasonable to conclude that the emergence of novel pathogens happens over time scales of years and decades (i.e. these are not rare events) and it is happening as fast as ever, if not faster, in the early twenty-first century.

More detailed characterization of the drivers of emergence events and more mechanistic explanations of the emergence process based on in-depth investigation are few and far between. Possible examples (exhibiting different levels of rigour) include the origins of HIV-1, BSE, SARS coronavirus, H5N1 influenza A and Nipah virus [56]. The origin of each of these pathogens is a unique and complex story and does not immediately suggest obvious generalizations. Nonetheless, although the emergence of a specific pathogen may always be essentially unpredictable, patterns are discernable [54]. The challenge is to move beyond statements that may well be correct but have little practical value, such as ‘new pathogens will emerge’, towards more informative (quantitative) predictions of how often this might happen, where it is likely to happen, and whether it is likely to represent a serious threat to human or animal health. This kind of information would be of considerable practical value, for example, in helping determine what kinds of disease surveillance systems are needed and where they should be deployed [52]. Meeting the challenge requires a better (and more quantitative) understanding of the drivers of pathogen emergence than exists at present.

## 6. The future

The kinds of formal, quantitative analysis described here can be valuable guides towards answering questions such as how probable a disease outbreak is, how far and how fast it will spread, and how best to control it. But how much reliance should be placed on the outputs of these analyses? One area where quantitative analysis and prediction have been highly successful in informing policy is climate change modelling. In this context, the inherent uncertainties in making predictions about a complex and incompletely understood system are well recognized and widely discussed. There are a substantial number of climate change models, and these agree in some respects and disagree in others [57]. It is not expected that the models will be correct in every detail; what is expected is that model predictions can be considered a reasonable basis for action by stakeholders. The same applies, over much shorter time-scales, to weather forecasting: we do not expect the weather forecast to be precisely correct on every occasion we look at it, but we do expect it to be sufficiently accurate often enough to be useful. ‘A reasonable basis for action’ seems a sensible aspiration for predictions about future disease risks.

One way to help judge whether a particular analysis constitutes a reasonable basis for action (i.e. that it is policy-ready as opposed to merely policy-relevant) is to be able to point to commonly accepted professional standards. A recently published guide to good practice for quantitative analysis of epidemiological data [58] is intended to be helpful `to users of the outputs, particularly funders and policy-makers, to assist them in making an informed assessment (recognizing that needs, expectations and standards evolve through time). The kinds of issues that are addressed include: clarity of objectives, transparency, good documentation and record keeping, verification, validation, the role of peer review and audit, reproducibility, and clear and accurate communication of outputs, assumptions and uncertainties. Indeed, an important advantage of formal quantitative models over informal approaches to prediction is that the models' inputs, assumptions and logical structure are set out explicitly, allowing them to be criticized and modified as appropriate [59]. Informal approaches, such as those based on expert opinion, tend to be much less transparent.

Nonetheless, while the techniques available for quantitative analysis become ever more sophisticated, useful predictions of future disease risk still depend ultimately on the quality and the availability of input data. Often, this is a severe limitation. In some cases, this reflects a genuine lack of data but in others it reflects an unwillingness to share data. The latter is a serious issue at the international level, with some countries sometimes unwilling to report disease data to the World Health Organisation (WHO) or the World Organisation for Animal Health (OIE). Implementation of the 2005 International Health Regulations should improve the situation, at least for human diseases, but the underlying issue is for the international community to provide incentives for reporting that compensate for the often severe disincentives associated with disease outbreaks, such as restrictions on travel and trade [52]. Even at the national level, government departments, public and animal health agencies, or academic institutes may be unwilling to release data on disease, host or drivers, thereby relinquishing ‘ownership’ of those data. Since the timely flow of usable information is an essential part of managing disease risks, not least when a human or animal health emergency arises, this problem needs to be addressed, noting that it is a cultural issue rather than a technical one. To do so, it is important that responsibilities for data curation, quality control and dissemination are set out and that individuals are recognized and appropriately rewarded for these activities.

In conclusion, the application of formal, quantitative methods to predict future infectious disease risks has become both more sophisticated and more widely accepted in recent years. This subject is very much the domain of epidemiologists, but nonetheless draws heavily on other disciplines. Clinicians and biologists provide a detailed understanding of the disease process; demographers and behavioural scientists provide knowledge of host populations; a wide variety of disciplines—e.g. climatology, agricultural economics, trade law, urban planning and food science—contribute to understanding drivers of disease risks; public and animal health workers, and also sociologists, provide understanding of the implementation of and compliance with intervention measures; and mathematicians, statisticians and computer scientists provide the analytical tools for data management, analysis and modelling. An important challenge for the future is to develop frameworks for integrating these disparate kinds of activity to provide more reliable and useful predictions of future disease risks.
